# Supplementary material for: Enhancing Soybean Salt Tolerance with GSNO and Silicon: A Comprehensive Physiological, Biochemical, and Genetic Study
Source: Int J Mol Sci. 2025 Jan 13;26(2):609. doi: 10.3390/ijms26020609 (PMC11765656; doi:10.3390/ijms26020609)
Supplement: Supplementary file 1 [file ijms-26-00609-s001.zip › ijms-3324030-supplementary.pdf]

Enhancing Soybean Salt Tolerance with GSNO and Silicon: A Comprehensive Physiological, Biochemical, and Genetic Study

Meshari Winledy Msarie [1,†], Nusrat Jahan Methela [2,3,†], Mohammad Shafiqul Islam [2,3], Tran Hoang An [1], Ashim Kumar Das [2], Da-Sol Lee [2], Bong-Gyu Mun [4] and Byung-Wook Yun [1,2,*]

[1] Department of Food Security and Agricultural Development, College of Agriculture and Life Sciences, Kyungpook National University, Daegu 41566, Republic of Korea
[2] Department of Applied Biosciences, College of Agriculture and Life Sciences, Kyungpook National University, Daegu 41566, Republic of Korea; methela.ag@nstu.edu.bd (N.J.M.); shafik.ag@nstu.edu.bd (M.S.I.)
[3] Department of Agriculture, Noakhali Science and Technology University, Noakhali 3814, Bangladesh
[4] Department of Environmental and Biological Chemistry, Chungbuk National University, Cheongju 28644, Republic of Korea; munbg@cbnu.ac.kr
* Correspondence: bwyun@knu.ac.kr
† These authors contributed equally to this work.

Table S1: primer list used for the study

Gene name	Gene ID	Forward Primer	Reverse Primer
GmActin7	GLYMA_06G150100	GCAAGAACTCGAGACTGCAA	CCAGCAGCTTCCATTCCAAT
GmNHX1	GLYMA_10G158700	TGATTGGTGTTTGCACTGGC	TTCACCTGAAACCCGGCATT
GmSOS2	GLYMA_13G166100	TCCCCAACTCAAATCTGTGAAG	GAAGGTTCCGTGGCCTAACA
GmNIP2.1	GLYMA_09G238200	TCATCTTCCCCTGGCTCTCT	TAGCCCAGCAGAACCACTTC
GmNIP2.2	GLYMA_18G259500	CATCTCAGGGGCACACATGA	CTGATGGCCGTAACAGCTCT
GmAKT1	GLYMA_06G071100	TGCAGCTTCCAAAGGCAATG	TCACGGATTCATGCCTACCC
GmLbR	GLYMA_07G232900	CTGCTGGTAGGACCCCATTC	GCAACACCATCTTCTTCCGC

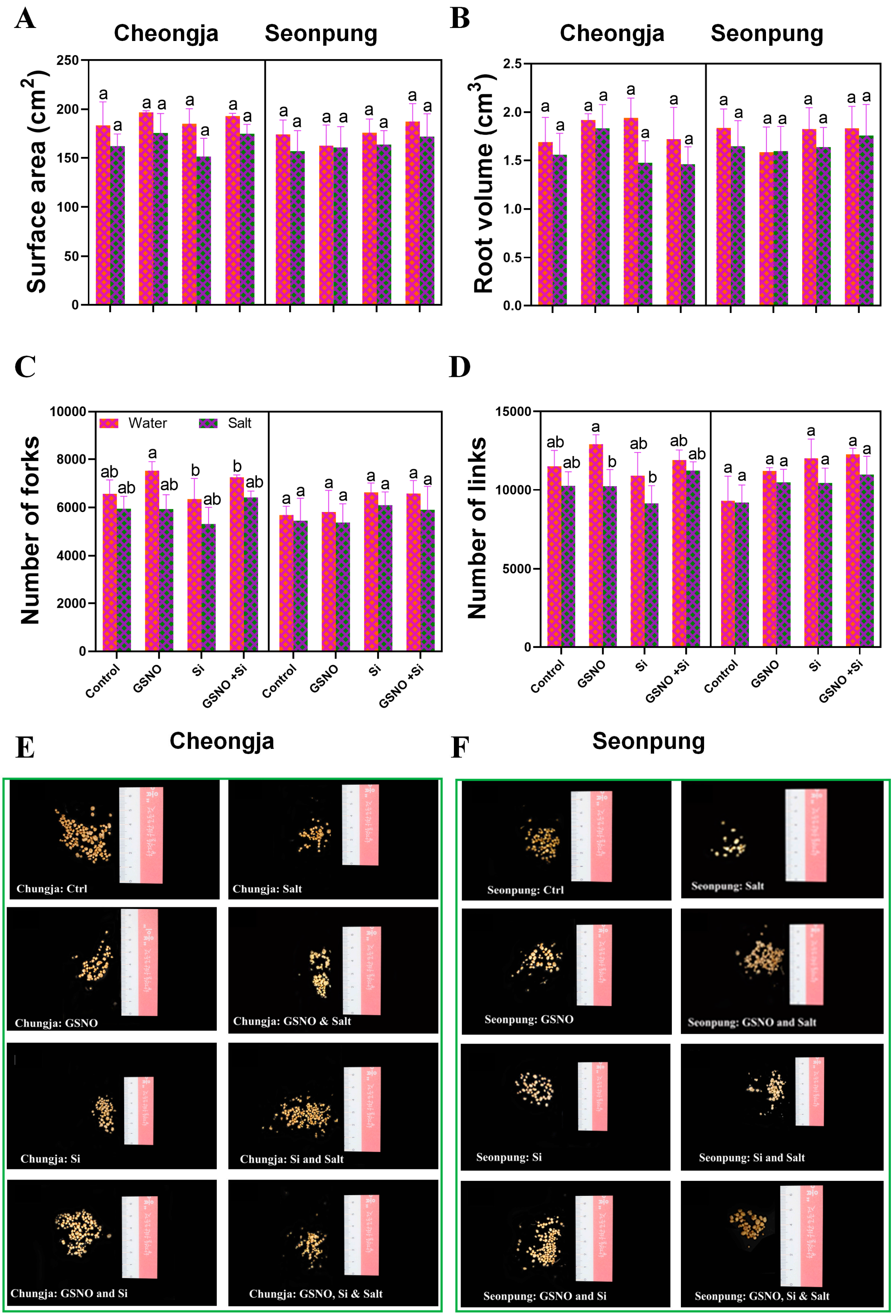
A
Cheongja
Seonpung
Surface area (cm^2)
B
Cheongja
Seonpung
Root volume (cm^3)
C
Water
Salt
Number of forks
D
Number of links
Control
GSNO
Si
GSNO +Si
E
Cheongja
Chungja: Ctrl
Chungja: Salt
Chungja: GSNO
Chungja: GSNO & Salt
Chungja: Si
Chungja: Si and Salt
Chungja: GSNO and Si
Chungja: GSNO, Si & Salt
F
Seonpung
Seonpung: Ctrl
Seonpung: Salt
Seonpung: GSNO
Seonpung: GSNO and Salt
Seonpung: Si
Seonpung: Si and Salt
Seonpung: GSNO and Si
Seonpung: GSNO, Si & Salt

Figure S1. Combined application of GSNO and Si affected root characters including nodules in soybean variety- seonpung and chungja under salt stress- (A) surface area, (B) root volume, (c) number of root forks, (D) number of root links, (E) morphological view of nodules in cheongja, and (F) morphological view of nodules in seonpung. The bar graph displays the standard error of the mean, with each data point representing the average of three replicates. Letters on the bars indicate significant differences determined by Duncan's Multiple Range Test (DMRT) at a significance level of 5% ($p \leq 0.05$). Same letters represent no significant differences.

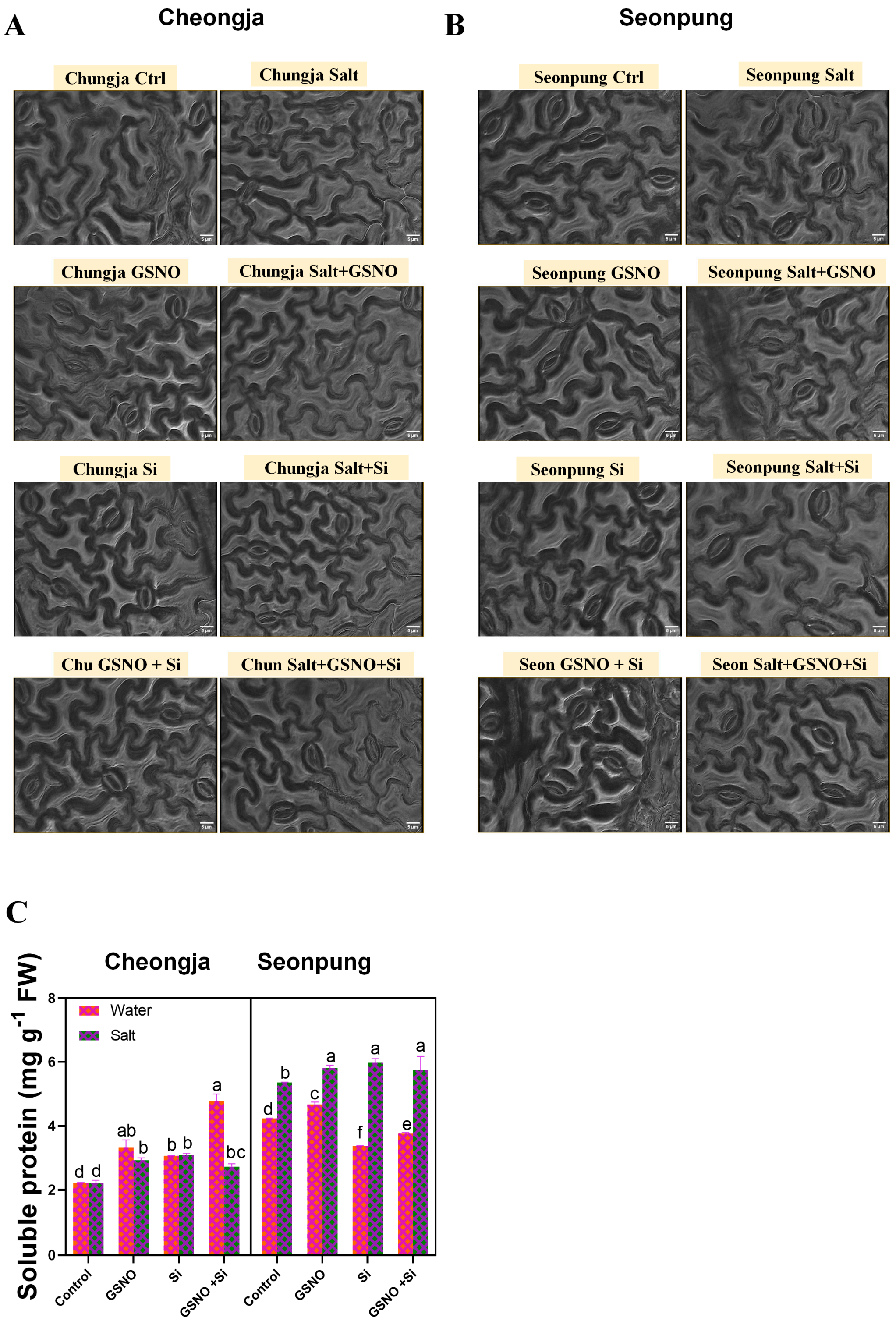

Figure S2. Combined application of GSNO and Si improved stomatal aperture in soybean variety-seonpung and chungja under salt stress- (A) microscopic view of stomata in cheongja, (B) microscopic view of stomata in seonpung and (C) soluble protein content. Bar scale indicates 5μm. The bar graph displays the standard error of the mean, with

each data point representing the average of three replicates. Letters on the bars indicate significant differences determined by Duncan's Multiple Range Test (DMRT) at a significance level of 5% (p ≤ 0.05). Same letters represent no significant differences.

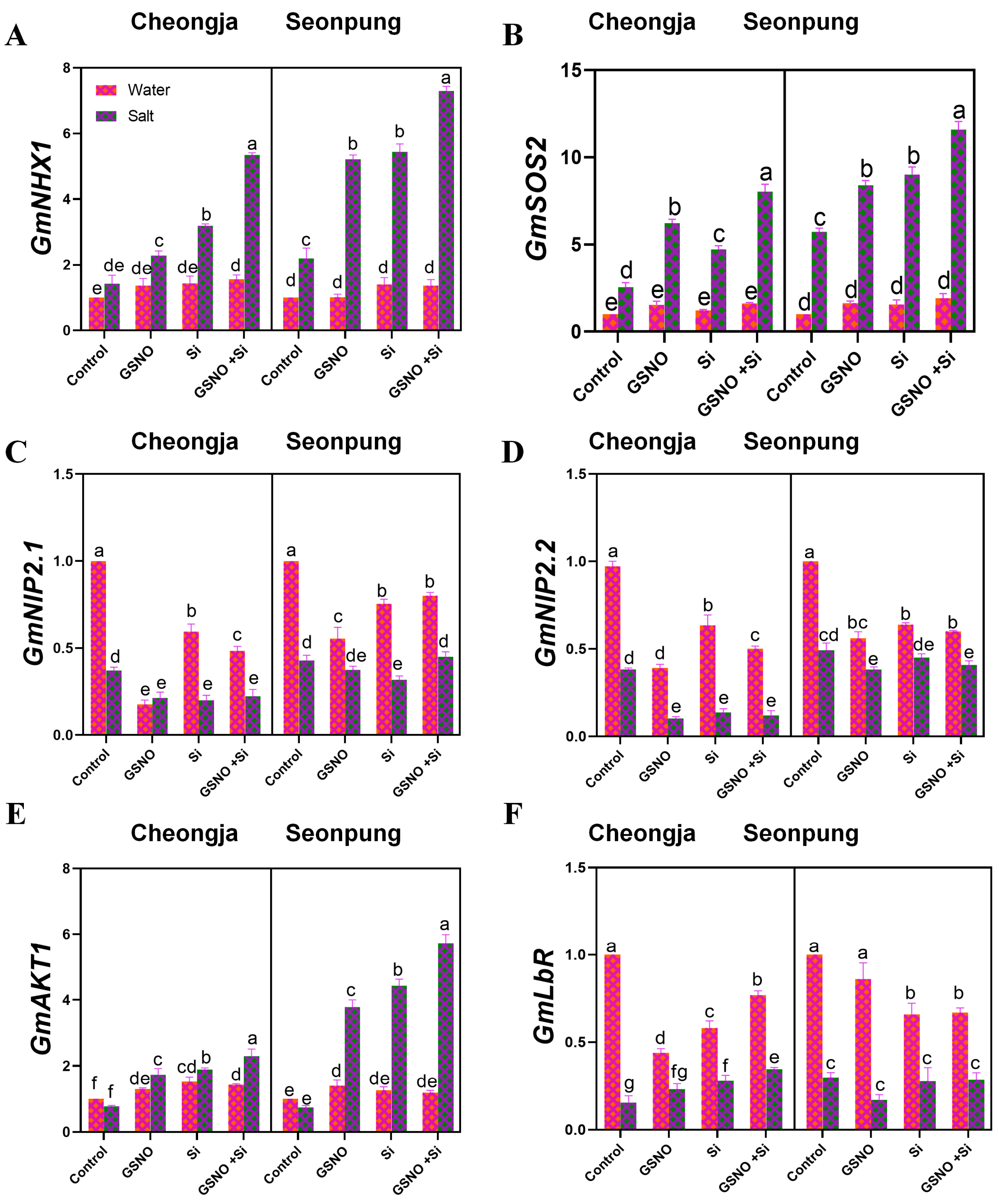

Figure S3. Combined application of GSNO and Si enhanced relative gene expression in soybean cultivar-seonpung and cheongja under salt stress- A) *GmNHX1*, B) *GmSOS2*, C) *GmNIP2.1*, D) *GmNIP2.2*, E) *GmAKT1* and F) *GmLBR1*. The bar graph displays the standard error of the mean, with each data point representing the average of three replicates. Letters on the bars indicate significant differences determined by Duncan's Multiple Range Test (DMRT) at a significance level of 5% ($p \leq 0.05$). Same letters represent no significant differences.
